# Validation of the global limb anatomical staging system in Vietnamese patients treated for chronic limb-threatening ischemia

**DOI:** 10.1186/s42155-024-00433-x

**Published:** 2024-03-05

**Authors:** Tran Minh Bao Luan, Nguyen Huu Tuong, Tran Ngoc Dang, Do Dang Khoa

**Affiliations:** 1 Department of Cardiovascular and Thoracic Surgery, Faculty of Medicine, University of Medicine and Pharmacy at Ho Chi Minh City, Ho Chi Minh City, 700000 Viet Nam; 2 Deparment of Thoracic and Vascular Surgery, University Medical Center HCMC, Ho Chi Minh City, 700000 Viet Nam; 3 Department of Adult Cardiovascular Surgery, University Medical Center HCMC, Ho Chi Minh City, 700000 Viet Nam; 4 Faculty of Public Health, University of Medicine and Pharmacy at Ho Chi Minh City, Ho Chi Minh City, 700000 Viet Nam

**Keywords:** Global limb anatomical staging system, Chronic limb-threatening ischemia, Endovascular intervention, Peripheral artery disease

## Abstract

**Background:**

Chronic limb-threatening ischemia (CLTI) is the most severe clinical form of peripheral artery disease (PAD), accounting for approximately 11%, and is strongly associated with the incidence of amputation, cardiovascular events, and mortality. The Global Vascular Guideline (GVG) proposed a new Global Anatomic Staging System (GLASS) for evaluating the anatomic complexity of arterial lesions. However, more research is required to evaluate outcomes after endovascular intervention in CLTI patients using the GLASS.

**Objective:**

Our study aimed to describe clinical characteristics, arterial lesions, and endovascular interventions according to three grades of GLASS in the Vietnamese population. We evaluated the technical success, mortality rate, and probability to preserve the limb according to the GLASS.

**Methods:**

All patients were diagnosed with CLTI and underwent infrainguinal endovascular intervention at the Department of Thoracic and Vascular Surgery, University Medical Center, Ho Chi Minh City from June 2020 to June 2022. All patients were evaluated before intervention and follow-up at 6 and 12 months after intervention. Patients were divided into three groups according to the GLASS, thereby comparing the technical success, mortality, and amputation rates. This retrospective study describes a series of cases.

**Results:**

The study sample evaluated 82 lower limbs of 82 patients, in which GLASS class I, II, and III lesions accounted for 36.6%, 43.9%, and 19.5% of the patients, respectively. The rates of technical success in the groups gradually decreased according to the complexity of the lesions (90%, 86.11%, and 56.25% for GLASS I, II, and III, respectively; *p* = 0.012). Notably, limb-based patency (LBP) at 12 months was significantly lower in the GLASS III group than in the GLASS I and II groups (22.22% vs 88.89% and 67.74%, respectively; *p* = 0.001). The amputation rates at 12 months in GLASS groups I, II, and III were 13.3%, 22.2%, and 50%, respectively (*p* = 0.021), while the mortality rates at 12 months were 0%, 8.33%, and 25%, respectively (*p* = 0.015).

**Conclusion:**

In patients with CLTI of higher GLASS stages, the rates of technical success were lower and the amputation and mortality rates were higher.

**Supplementary Information:**

The online version contains supplementary material available at 10.1186/s42155-024-00433-x.

## Background

Peripheral artery disease (PAD) of the lower extremities is a growing global healthcare problem, estimated to affect over 230 million adults worldwide [1]. Chronic limb-threatening ischemia (CLTI) is an end-stage PAD, and accounts for approximately 11% of patients with PAD [2]. According to the Global Vascular Guidelines (GVG) published in 2019, CLTI is a clinical syndrome that occurs in patients with PAD who have symptoms of ischemic resting pain and tissue loss (ulceration or gangrene) in the lower limb lasting more than 2 weeks, excluding causes due to venous diseases, trauma, emboli, or non–atherosclerotic etiologies [3]. It is strongly associated with amputation, cardiovascular events, and mortality [4]. The prevalence and complexity of CLTI is increasing due to longer life expectancy and increase of coexisting conditions such as diabetes, dyslipidemia, smoking, and lack of exercise [1]. Although there have been advances in the treatment of patients with CLTI, the amputation rate remains high at approximately 20% within 12 months, while the mortality rate is nearly 50% within 5 years of diagnosis [5–8].

Revascularization treatment with bypass surgery or endovascular intervention is the treatment in patients with CLTI aiming to restore blood flow and preserve the limb [2, 9, 10]. In 2019, to aid clinical decision-making in everyday practice and facilitate future research, the GVG proposed the PLAN concept, including patient risk estimation, limb staging, and the anatomic pattern of disease [3]. In addition, the Wound, Ischemia, and Foot Infection (WIfI) scoring system has been used to evaluate limb staging [3]. Regarding the anatomical pattern, the Trans-Atlantic Inter-Society Consensus (TASC) scoring system was previously used, which classified the severity of vascular lesions into TASC A, B, C, and D based on the increasing complexity of the following three separate lower limb vascular levels: aortoiliac, femoropopliteal, and infrapopliteal arteries [11]. Based on expert consensus for patients undergoing infrainguinal endovascular intervention, the GVG recently introduced a new classification system called the Global Limb Anatomical Staging System (GLASS) [3]. The GLASS introduced the concepts of target artery path (TAP) and limb-based patency (LBP) to assess the success and maintenance of blood flow after intervention. It has three levels of complexity (I, II, and III), which helps predict technical success and LBP after endovascular intervention and is expected to correlate with clinical outcomes in patients with CLTI [3, 12].

At our center, for GLASS III lesions, patients with low perioperative risk are still given bypass surgery first. However, the choice of treatment methods depends on the patient’s economic status and preferences. In Vietnam, with the development of instruments, techniques, and the experience of vascular surgeons, endovascular intervention is becoming more popular for treating CLTI, even with complex anatomical lesions such as TASC C and D [13, 14]. Many centers, including Cho Ray Hospital, the University Medical Center of Ho Chi Minh City, and Viet Duc Hospital, have applied this technique [13, 14]. Currently, many studies in Vietnam have evaluated the results of endovascular intervention for PAD at individual anatomical levels, including the aortoiliac, femoropopliteal, and infrapopliteal arteries, using the TASC scoring system [13, 14]. However, no study has investigated the application of GLASS and its relationship with the results of endovascular interventions in the infrainguinal arteries in Vietnam. Therefore, this study aimed to evaluate the technical success, mortality rate, and ability to preserve the limb according to the GLASS in the Vietnamese population.

## Materials and methods

### Study design

This retrospective review includes a series of cases conducted at the Thoracic and Vascular Surgery Department of the University Medical Center of Ho Chi Minh City.

The study was approved by the local institutional ethics committee of Ho Chi Minh University of Medicine and Pharmacy, opinion 448/HĐĐĐ – ĐHYD. The informed consent was waived. We did not receive funding from any individual or organization. All steps followed the STROBE checklist (see Supplementary Material for details).

### Data collection

All patients were diagnosed with CTLI at the Department of Thoracic and Vascular Surgery of the University Medical Center of Ho Chi Minh City between June 2020 and June 2022. The inclusion criteria were as follows: diagnosis of CLTI, treatment with infrainguinal endovascular intervention according to the GVG, and the existence of complete medical records and clinical, laboratory, and interventional protocol information.

The exclusion criteria were as follows: patients with > 50% stenosis or occlusion due to aortoiliac diseases, patients previously treated with infrainguinal bypass surgery or hybrid interventions on the same limbs, or patients with other associated pathologies such as trauma, vasculitis, Buerger's disease, venous disorders, and connective tissue disorders.

**Fig. 1:**
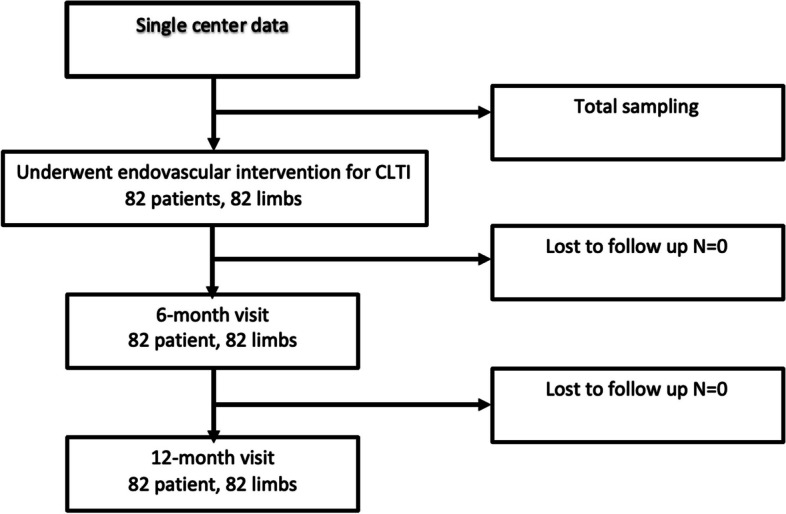
Study flow diagram

During the 2-year period of the study, we had a total of 338 cases of PAD. Of which 183 cases were bypass surgery and 155 cases were general endovascular intervention. There were 82 cases of infrainguinal endovascular intervention to treat CLTI, patients had complete information from hospital admission to follow-up examination. Using total sampling method, we collected all 82 of these cases (Fig. 1). To avoid possible bias in data collection, well-trained staff, which were resident doctors studying and working at our department, collected all data using structured forms with predetermined measures (see Supplementary Material for details). Endovascular intervention procedures were performed by four surgeons. All surgeons have over 10 years of experience, have been trained in endovascular intervention abroad and were certified in endovascular intervention to treat PAD.

### Procedure

Please see the Supplementary Material for details.

### Measures

The concept underlying the GLASS is that there is a continuous artery running from the aortoiliac level down to the foot, called the TAP, and endovascular intervention for CLTI requires TAP identification. The GLASS is based on the stenosis or occlusion of the femoropopliteal and infrapopliteal arteries, including stage I, II, and III. The inframalleolar arteries is classified into P0, P1, P2 (please see Supplementary Material for details). The patients (limbs) were divided into three groups according to the GLASS classification (I, II, and III) based on digital subtraction angiography. The variables were evaluated preoperatively, intraoperatively, and postoperatively.

The preoperative variables included baseline characteristics and risk factors, while the intraoperative variables included the Ankle Brachial Index (ABI) before intervention, anesthesia methods, location of lesions, access positions, intervention time, endovascular intervention methods, technical success, and complications. The definition of technical success was the successful completion of the procedure, residual stenosis after intervention ≤ 30%, no artery dissection [15, 16], and the creation of a continuous arterial pathway from the aortoiliac level down to the common femoral artery, superficial femoral artery, popliteal artery, and at least one branch of the infrapopliteal level (anterior tibial artery, posterior tibial artery, or peroneal artery) to the foot [3]. Technical failure was defined as the inability to pass the guidewire across the lesion, advance the balloon across the lesion, or create good flow after endovascular intervention.

The postoperative outcomes included major limb amputation, mortality, ABI changes, and LBP at 6 and 12 months postoperatively across the GLASS stages. Major limb amputation was defined as any above-ankle amputation. Duplex ultrasound was used as the main follow-up imaging study, and repeat angiography was performed if a re–intervention was planned. LBP was defined as the absence of any of the following: (1) occlusion or critical stenosis (> 70%) within the TAP on the imaging study, (2) reintervention affecting any portion of the TAP, or (3) evidence of hemodynamic compromise (> 50% stenosis in the TAP or a decrease in ABI of ≥ 0.15) with ongoing clinical symptoms of CLTI in the index limb.

### Statistical analysis

Data analysis was performed using descriptive statistics using Stata 14.0 software. Quantitative variables, including the ABI and intervention time, are described as means ± standard deviations or medians with interquartile ranges. In contrast, categorical variables such as baseline characteristics, risk factors, anesthesia methods, locations of lesions, access positions, endovascular intervention methods, technical success, complications, major limb amputation, and mortality are described as numbers and percentages. Statistical analyses included the Chi-square or Fisher's exact tests (if expected frequency < 5) for comparisons of proportions and ANOVA or the Kruskal-Wallis test for comparisons of means between groups, with a significance level of *p* < 0.05.

## Results

A total of 82 patients (82 limbs) underwent endovascular intervention. The numbers of limbs classified as GLASS I, II, and III were 30, 36, and 16, respectively. The GLASS III rate was lower than the GLASS I, II. Because patients in GLASS III group tend to choose bypass surgery first, when discussing about the technical failure rate of endovascular intervention.

### Baseline characteristics and risk factors

The baseline characteristics and risk factors are described in Table 1. Of these factors, only coronary artery disease and heart failure showed statistically significant differences between the groups (*p* < 0.05). This facilitated the comparison of results from endovascular intervention treatments among the three GLASS classification groups.

**Table 1:** General clinical characteristics of 82 limbs with CLTI by different GLASS stages

	GLASS I *n* = 30 (%)	GLASS II *n* = 36 (%)	GLASS III *n* = 16 (%)	*p*
Age	69.47 ± 6.5	73.17 ± 11.23	76.24 ± 11.4	0.06**
Male	20 (66.67)	24 (66.67)	8 (50)	0.463*
Smoking	12 (40)	20 (55.56)	8 (50)	0.45*
Diabetes	18 (60)	30 (83.33)	9 (56.25)	0.054*
Hypertension	20 (66.67)	32 (88.89)	14 (87.5)	0.056*
Coronary artery disease	8 (26.67)	22 (61.11)	6 (37.5)	0.016*
Heart failure	2 (6.67)	14 (38.89)	6 (37.5)	0.007*
Chronic kidney disease	4 (13.33)	8 (22.22)	2 (12.5)	0.547*
Cerebrovascular disease	2 (6.67)	2 (5.56)	1 (6.25)	0.982*
Dyslipidemia	12 (40)	18 (50)	12 (75)	0.076*

^**^Kruskall-Wallis test

^*^χ^2^test

### Intraoperative characteristics

All procedures were performed under local anesthesia. The characteristics of the lesions and endovascular intervention methods in the three GLASS groups are shown in Table 2. The lesions in all three groups were located at either one or both of the femoropopliteal and infrapopliteal levels. Due to objective conditions of instruments and economic conditions of the patients. all intervention cases used conventional balloons and stents, not drug-coated balloons or drug-eluting stents. The access sites were mostly via the ipsilateral common femoral artery in 76/82 limbs. Notably, the intervention time was significantly longer in the GLASS III group than in the other two groups, with a mean intervention time of 79.69 ± 17.2 min (*p* < 0.05). The technical success rates in the GLASS I, II, and III groups were 90%, 86.11%, and 56.25%, respectively, with a statistically significant difference (*p* < 0.05). For cases of unsuccessful intervention, we considered bypass surgery, or medical treatment, or minimal amputation and wound care, depending on the patient’s overall condition and preferences.

**Table 2:** Intraoperative characteristics classified according to the GLASS stages

	GLASS I *n* = 30 (%)	GLASS II *n* = 36 (%)	GLASS III *n* = 16 (%)	*P*
**Location of lesions**				
FP	12 (40.0)	4 (11.1)	0 (0)	< 0.001*
IP	14 (46.7)	8 (22.2)	4 (25)	
Both FP and IP	4 (13.3)	24 (66.7)	12 (75)	
**Infra-malleolar perfusion P0 or P1**	28 (83.3)	34 (94.4)	11 (68.8)	0.015*
**Access position**				
Ipsilateral CFA	30 (100)	34 (94.4)	12 (75)	0.007*
Contralateral CFA	0 (0)	2 (5.6)	4 (25)	
**Intervention time (minutes)**	59.73 ± 19.1	70 ± 35.2	79.69 ± 17.2	0.033***
**ABI before intervention**	0.332 ± 0.22	0.32 ± 0.26	0.13 ± 0.18	0.013***
**Technical success**	27 (90)	31 (86.11)	9 (56.25)	0.012*
**Endovascular intervention method**				
Balloon angioplasty	16	12	5	0.183*
Balloon angioplasty and stenting	12	22	9	

*FP* Femoropopliteal, *IP* Infrapopliteal, *CFA* Common femoral artery

^*^χ^2^test

^***^ANOVA test

Regarding the complications of endovascular intervention (Table 3), there was a higher trend of incidence in the GLASS III group; however, due to an insufficient number of events, most of the variables related to complications had a *p*-value > 0.05. Therefore, there was no statistically significant difference between the three GLASS groups. For cases of arterial dissection complications that slow the flow, if the lesions are located at the femoropopliteal level, we will proceed to place a stent at the dissection sites. If the lesions are located at the infrapopliteal level, we will try to dilate the baloon again to open up the true lumen.

**Table 3:** Complications in endovascular intervention

	GLASS I *n* = 30 (%)	GLASS II *n* = 36 (%)	GLASS III *n* = 16 (%)	p*
Distal embolisation	0 (0)	1 (2.78)	1 (6.25)	0.418
Bleeding, hematoma at the access site	2 (0)	2 (5.56)	2 (12.5)	0.665
Arterial dissection	3 (10)	5 (13.89)	6 (37.5)	0.049
Vascular perforation	0 (0)	0 (0)	1 (6.25)	0.124
Arteriovenous fistula	0 (0)	0 (0)	0 (0)	
Pseudoaneurysm	0 (0)	0 (0)	0 (0)	
Total complications	5 (16.67)	8 (22.22)	10 (62.5)	

^*^χ^2^test

### Postoperative characteristics

Because of objective conditions of facilities, currently our center can not apply toe pressure measurement in the diagnosis and treatment of PAD. We assessed the improvement in the ABI, LBP, amputation rate, and mortality rate at 6 and 12 months after the intervention. We found that there were no cases requiring re–intervention within 12 months of successful intervention.

There was a trend of an increased ABI after the endovascular intervention; however, there was no statistically significant difference in hemodynamic success (defined as an ABI increase of > 0.15) between the three GLASS groups (*p* > 0.05). In contrast, the maintenance of perfusion in the TAP at 6 and 12 months decreased as the GLASS stage increased, with a statistically significant difference (*p* < 0.05). Moreover, the 12-month LBP was significantly lower in the GLASS III group than in the GLASS I and II groups (22.22% vs. 88.89% and 67.74%, respectively; *p* < 0.05) (Tables 4 and 5).

**Table 4:** ABI and Duplex ultrasound imaging studies after the successful technical intervention

	GLASS I *n* = 27 (%)	GLASS II *n* = 31 (%)	GLASS III *n* = 9 (%)	p**
ABI after intervention	0.7 ± 0.17	0.58 ± 0.14	0.61 ± 0.16	0.016***
Increase in ABI	0.26 ± 0.1	0.29 ± 0.16	0.38 ± 0.12	0.71***
ABI increase ≥ 0.15 (Number of cases)	26 (96.3%)	30 (96.77%)	7 (77.78%)	0.086*
ABI after 6 months	0.68 ± 0.26	0.55 ± 0.17	0.49 ± 0.19	0.025***
ABI after 12 months	0.64 ± 0.22	0.5 ± 0.25	0.47 ± 0.13	0.037***
Maintenance of perfusion of the TAP after 6 months (Number of cases)	27 (100)	25 (80.65)	5 (55.56)	0.003*
Maintenance of perfusion of the TAP after 12 months (Number of cases)	24 (88.89)	21 (67.74)	2 (22.22)	0.001*

^*^χ^2^ test

^***^ANOVA test

**Table 5:** Amputation rate and mortality rate at 6 months and 12 months after the intervention

	GLASS I *n* = 30 (%)	GLASS II *n* = 36 (%)	GLASS III *n* = 16 (%)	p*
Amputation at 6 months	4 (13.3)	8 (22.2)	8 (50)	0.021
Amputation at 12 months	4 (13.3)	8 (22.2)	8 (50)	0.021
Mortality at 6 months	0 (0)	0 (0)	1 (6.25)	0.015
Mortality at 12 months	0 (0)	3 (8.33)	4 (25)	0.015

^*^χ^2^test

During the perioperative period, we did not record any mortality or limb amputations. Regarding the amputation rate 6 months postoperatively, 20 patients underwent amputation, of which the GLASS III group had the highest rate, accounting for 50% of the patients (*p* < 0.05). However, at 12 months, no additional cases of amputation were recorded. Notably, the mortality rate increased when the GLASS stage was higher (*p* < 0.05). In total, no deaths occurred in the GLASS I group. After 12 months, the mortality rates in the GLASS I, II, and III groups were 0%, 8.33%, and 25%, respectively, with a statistically significant difference (*p* < 0.05).

## Discussion

Our study aimed to evaluate the technical success, mortality rate, and ability to preserve the limb according to the GLASS in a Vietnamese population. In our study, we found that the GLASS stage was significantly associated with technical failure, mortality, and amputation rates.

Only a small number of studies have evaluated the efficacy of the GLASS in the treatment of CLTI. In 2020, Tokuda et al. published a study on the prediction of the technical success of endovascular intervention in patients with CLTI using the GLASS [17]. Their retrospective study was conducted at a single center with 400 lesions in 257 patients. These 400 lesions were divided into three groups according to the GLASS stages and compared for patient characteristics, lesion characteristics, technical success, and complications in each group. They concluded that in GLASS III, anatomic/limb severity was more complex and procedure complications were more frequent. The GLASS reliably predicts the technical success of de novo TAP in patients with CLTI [17]. Similarly, El Khoury et al. published a study evaluating the correlation between the GLASS stages and outcomes after infrainguinal revascularization for CLTI, including 194 limbs of 167 patients. They concluded that GLASS III stage was strongly associated with major adverse clinical outcomes after revascularization in patients with CLTI [18]. In 2022, Shirasu et al. published a meta-analysis on the predictive ability of the GLASS for technical success and limb status after revascularization [19]. Their study included seven cohort studies and one randomized controlled trial with a sample size of 2,483 limbs from 2,204 patients. They performed subgroup analyses of endovascular intervention and found that the amputation-free survival, limb salvage rate, and major adverse limb events were significantly affected by advanced GLASS stages. They concluded that GLASS assists in both the prediction of technical failure and LBP after endovascular intervention and the differentiation of the risk of limb-related outcomes, and therefore recommended that more research on the outcomes of bypass surgery and endovascular intervention in advanced GLASS stages be performed [19].

Regarding the technical failure rate, the GLASS constitutes a paradigm shift in the anatomical evaluation of CLTI, evolving from a lesion-based to a limb-based approach [3]. In our study, nearly 50% of the limbs had combined femoropopliteal and infrapopliteal diseases, which allowed for a general assessment of complex multilevel disease and the prediction of technical failure after endovascular intervention. In the present study, the technical failure rates of the GLASS I, II, and III groups were 10%, 13.89%, and 43.75%, respectively, whereas Shirasu et al. reported technical failure rates of 3.9%, 5.3%, and 27.9%, respectively [19]. Regarding the complications of endovascular intervention, there was a higher incidence in the GLASS III group; however, we found no statistically significant difference between the three GLASS groups. We also found no statistically significant difference in complications of distal embolization, and vascular perforation between the three groups, and there were no arteriovenous fisula or pseudoaneurysms in our study. Bleeding and hematoma complications maybe more related to puncture site and introducer size. Notably, Tokuda et al. reported three cases of arteriovenous fistula in the GLASS III group, whereas we did not encounter any such cases in our study [17]. According to the personal experiences of surgeons at our center, in complex lesions, such as those classified as GLASS III, aggressive pushing sometimes had to be used for the guidewires to cross the calcified total occlusion, which led to more complications such as perforation and artery dissection. However, these were just personal experiences, without objective and specific quantification. Other studies have shown that inframalleolar GLASS is associated with major amputation, major adverse limb events, and wound healing [20, 21]. In our study, the 12-month LBP was significantly lower in the GLASS III group (22.22%) when compared with the GLASS I and II groups (*p* < 0.05). Notably, the technical failure rate and 12-month LBP in the GLASS III group were similar with the predictions of the GVG (technical failure rate > 20% and LBP < 50%) [3]. We have had no cases of re-intervention within 12 months of follow up. Some patients with recurrence of CLTI symptoms received re-intervention after 12 months, or patient did not agree to re-intervention and only received medical treatment.

In our study, the mortality and amputation rates significantly increased when the GLASS stage was higher (*p* < 0.05). A meta-analysis by Shirasu et al. showed that the amputation-free survival, limb salvage rate, and major adverse limb events were significantly affected by advanced GLASS stages; however, overall survival was not significantly affected [19]. In contrast, Tokuda et al. showed that the major amputation rates at 1 year in the GLASS I, II, and III groups were 7.7%, 5.3%, and 11.5%, respectively, and there were no significant differences among the three groups with regard to major amputation at 1 year and 30-day mortality [17]. Thus, we found that there were differences in the outcomes between studies. We attribute this difference to differences in study populations. In Vietnam, primary medical care still has some limitations. CLTI patients come to our tertiary hospital mostly with widespread limb necrosis, with many uncontrolled comorbidities. This affects the outcomes of treatment. According to the GVG, the GLASS is only one part of considering a patient’s CLTI treatment, and the prediction of clinical outcomes should be considered in the PLAN concept [3]. In the PLAN concept, patient conditions are estimated to determine candidacy for limb salvage as well as perioperative risk, and limb severity is also assessed based on the WIfI classification.

To our knowledge, our study is the first to assess the application of the GLASS to endovascular intervention treating infrainguinal CLTI within the Vietnamese population. Applying the GLASS to endovascular intervention for CLTI in Vietnamese patients may help develop a more systematic approach and predict further treatment results in the future.

Our study had some important limitations. First, this was a retrospective, single-center study; therefore, it may not accurately reflect other populations. Second, and most importantly, the number of lower limbs in the study was insufficient to evaluate and analyze the relationship between the GLASS and the outcomes of endovascular intervention. The proportion of the GLASS III group in our study was lower than that of GLASS I,II due to the tendency for GLASS III patients to have bypass surgery first. Therefore, perhaps the rate of comorbidities in the GLASS III group was lower and did not reflect reality, especially coronary artery disease and heart failure. Third, in addition to the complex lesions categorized as GLASS III, calcification and total occlusion of the vessels are confounding factors that may affect the technical success of the endovascular intervention. These were not investigated in our study and require multivariate analysis for more relevant results. The GLASS places more emphasis on lesion length than on the degree of stenosis; however, in clinical practice, approaching short segments of calcified or totally occluded vessels can also be challenging and lead to technical failure and higher complication rates. Therefore, further multicenter studies with larger sample sizes are warranted to more clearly analyze the treatment outcomes in Vietnamese patients.

## Conclusion

In patients with CLTI of a higher GLASS stage, the rate of technical success was lower and the amputation and mortality rates were higher.

### Supplementary Information

**Supplementary material 1.**

## Data Availability

The data that support the findings of this study are available from the authors but restrictions apply to the availability of these data, which were used under license from the University Medical Center of Ho Chi Minh City for the current study, and so are not publicly available. Data are, however, available from the authors upon reasonable request and with permission from the University Medical Center of Ho Chi Minh City.

## Abbreviations

CLTI: Chronic limb-threatening ischemia
GVG: Global Vascular Guideline
GLASS: Global anatomic staging system
LBP: Limb-based patency
PAD: Peripheral artery disease
TASC: Trans-Atlantic Inter-Society Consensus
TAP: Target artery path
ABI: Ankle brachial index
FP: Femoropopliteal
IP: Infrapopliteal
CFA: Common femoral artery
