# Supplementary material for: Validation of the global limb anatomical staging system in Vietnamese patients treated for chronic limb-threatening ischemia
Source: CVIR Endovasc. 2024 Mar 5;7:25. doi: 10.1186/s42155-024-00433-x (PMC10914645; doi:10.1186/s42155-024-00433-x)
Supplement: Supplementary file 1 — Supplementary material 1. [file 42155_2024_433_MOESM1_ESM.docx]

**Validation of Global Limb Anatomical Staging System in Vietnamese patients treated for chronic limb-threatening ischemia**

**Supplement**

1. **GLASS STAGING AND SURGICAL PROCEDURE**

The choice of endovascular intervention for the target artery path (TAP) according to the Global Limb Anatomical Staging System (GLASS) also depends on the inflow from the aortoiliac level and the outflow to the infra-malleolar level. Therefore, to predict the short-term, medium-term, and long-term success of endovascular intervention in maintaining the TAP and Limb-based patency (LBP) in patients with Chronic limb-threatening ischemia (CLTI) based on the levels of GLASS staging, the aortoiliac diseases must be corrected or have insignificant stenosis (<50%), and the patency of the foot arteries must be evaluated. The infra-malleolar target artery staging is as follows: P0 is a target artery crosses ankle into foot, with intact pedal arch; P1 is a target artery crosses ankle into foot, absent or severely diseased pedal arch; P2 is no target artery crossing ankle into foot.

**Table S1. GLASS Classification**

| **Score** | **Femoropopliteal** | | | | **Infrapopliteal** | | |
| --- | --- | --- | --- | --- | --- | --- | --- |
| 0 | Mild or no significant (<50%) disease | | | | Mild or no significant (<50%) disease | | |
| 1 | - Total length SFA disease <1/3 (<10cm) - May include single focal CTO (<5cm) as long as no flush occlusion - Popliteal artery with mild or no significant disease | | | | - Focal stenosis of tibial artery <3cm | | |
| 2 | - Total length SFA disease 1/3-2/3 (10-20cm) - May include CTO totaling <1/3 (10cm) but not flush occlusion - Focal popliteal artery stenosis <2cm, not involving trifurcation | | | | - Stenosis involving 1/3 total vessel length - May include focal CTO (<3cm) - Not including TP trunk or tibial vessel origin | | |
| 3 | - Total length SFA disease >2/3 (>20cm) length - May include any flush occlusion <20cm or non-flush CTO 10-20cm long - Short popliteal stenosis 2-5cm, not involving trifurcation | | | | - Disease up to 2/3 vessel length - CTO up to 1/3 length (may include tibial vessel origin but not TP trunk) | | |
| 4 | - Total length SFA occlusion>20cm - Popliteal disease>5cm or extending intro trifurcation - Any popliteal CTO | | | | - Diffuse stenosis >2/3 total vessel length - CTO>1/3 vessel length (may include vessel origin) - Any CTO of TP trunk if the Anterior tibial is not the target artery | | |
| **GLASS (I-III) Staging** | | | | | | | |
| **FEMOROPOPLITEAL** | | 4 | **III** | **III** | **III** | **III** | **III** |
|  |  | 3 | II | II | II | **III** | **III** |
|  |  | 2 | I | II | II | II | **III** |
|  |  | 1 | I | I | II | II | **III** |
|  |  | 0 |  | I | I | II | **III** |
|  |  |  | 0 | 1 | 2 | 3 | 4 |
|  | | **INFRAPOPLITEAL** | | | | | |

*(SFA: superficial femoral artery, TP: tibioperoneal, CTO: chronic total occlusion)*

Patients underwent endovascular intervention at cath lab. Below are digital subtraction angiography images showing pre- and post-intervention in the case of patient D.V.H, an 82-year-old male who was admitted to the hospital for dry gangrene of the first and second toes on the left foot. He underwent intervention on May 7th, 2022. (Medical ID: N22-0127018).


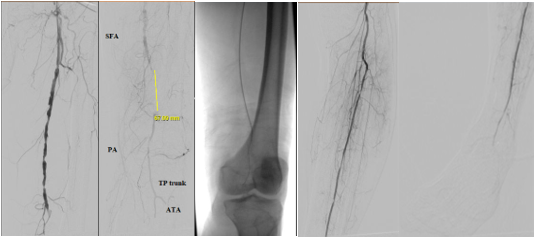


Figure S1. Moderate to severe, diffuse stenosis of 2/3 of the length of the SFA, with a total occlusion of 6.7 cm at the distal segment. The Popliteal artery PA and the TP trunk are not significantly narrowed. Lesion crossing was performed to access the P3 segment for imaging of the below-knee region to identify the target vessel, which is the anterior tibial artery. The lesion is classified as GLASS II, P1.


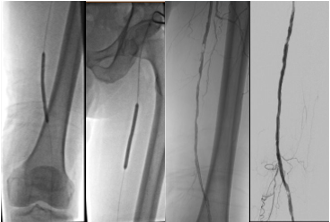


Figure S2. Balloon angioplasty of SFA lesions. Repeat imaging confirms residual stenosis of greater than 30% in the SFA. Stenting of the mid-section SFA.


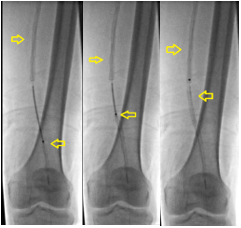


Figure S3. Additional stenting of the distal SFA


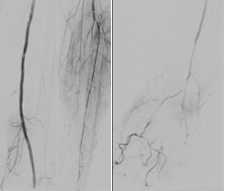


Figure S4. After revascularization, imaging shows a continuous arterial flow from the SFA down to the popliteal artery, the anterior tibial artery, and the ankle.

1. **DATA COLLECTION FORM**

All data were collected by well-trained staff using structured forms with predetermined measures.

**
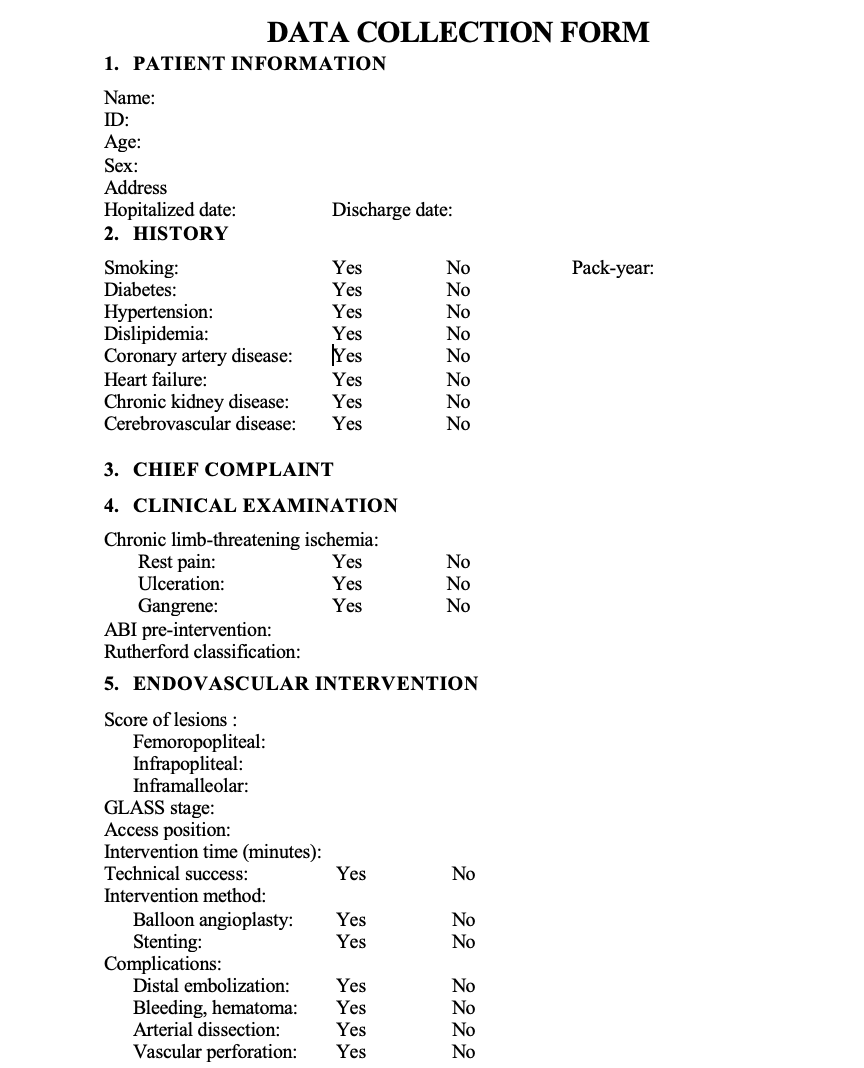
**


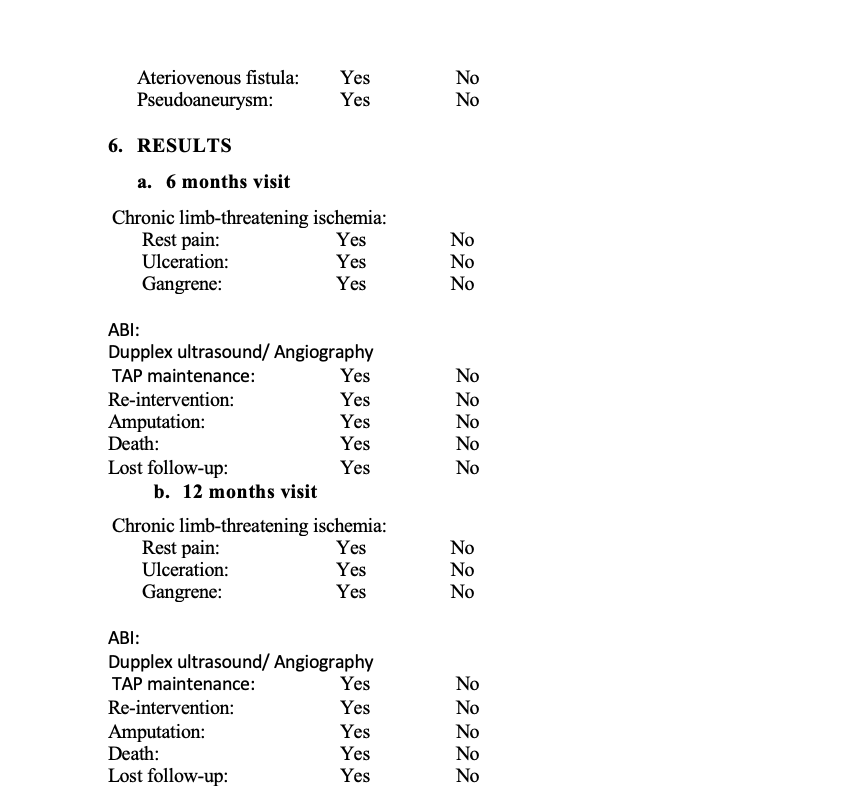


Figure S5: Data collection form.

1. **STROBE CHECKLIST**

All steps of our study were followed STROBE checklist

|  | Item No | Recommendation |  |
| --- | --- | --- | --- |
| **Title and abstract** | 1 | (*a*) Indicate the study’s design with a commonly used term in the title or the abstract |  |
|  |  | (*b*) Provide in the abstract an informative and balanced summary of what was done and what was found |  |
| Introduction | | |  |
| Background/rationale | 2 | Explain the scientific background and rationale for the investigation being reported |  |
| Objectives | 3 | State specific objectives, including any prespecified hypotheses |  |
| Methods | | |  |
| Study design | 4 | Present key elements of study design early in the paper |  |
| Setting | 5 | Describe the setting, locations, and relevant dates, including periods of recruitment, exposure, follow-up, and data collection |  |
| Participants | 6 | (*a*) Give the eligibility criteria, and the sources and methods of selection of participants |  |
| Variables | 7 | Clearly define all outcomes, exposures, predictors, potential confounders, and effect modifiers. Give diagnostic criteria, if applicable |  |
| Data sources/ measurement | 8* | For each variable of interest, give sources of data and details of methods of assessment (measurement). Describe comparability of assessment methods if there is more than one group |  |
| Bias | 9 | Describe any efforts to address potential sources of bias |  |
| Study size | 10 | Explain how the study size was arrived at |  |
| Quantitative variables | 11 | Explain how quantitative variables were handled in the analyses. If applicable, describe which groupings were chosen and why |  |
| Statistical methods | 12 | (*a*) Describe all statistical methods, including those used to control for confounding |  |
|  |  | (*b*) Describe any methods used to examine subgroups and interactions |  |
|  |  | (*c*) Explain how missing data were addressed |  |
|  |  | (*d*) If applicable, describe analytical methods taking account of sampling strategy |  |
|  |  | (*e*) Describe any sensitivity analyses |  |
| Results | | |  |
| Participants | 13* | (a) Report numbers of individuals at each stage of study—eg numbers potentially eligible, examined for eligibility, confirmed eligible, included in the study, completing follow-up, and analysed |  |
|  |  | (b) Give reasons for non-participation at each stage |  |
|  |  | (c) Consider use of a flow diagram |  |
| Descriptive data | 14* | (a) Give characteristics of study participants (eg demographic, clinical, social) and information on exposures and potential confounders |  |
|  |  | (b) Indicate number of participants with missing data for each variable of interest |  |
| Outcome data | 15* | Report numbers of outcome events or summary measures |  |
| Main results | 16 | (*a*) Give unadjusted estimates and, if applicable, confounder-adjusted estimates and their precision (eg, 95% confidence interval). Make clear which confounders were adjusted for and why they were included |  |
|  |  | (*b*) Report category boundaries when continuous variables were categorized |  |
|  |  | (*c*) If relevant, consider translating estimates of relative risk into absolute risk for a meaningful time period |  |
| Other analyses | 17 | Report other analyses done—eg analyses of subgroups and interactions, and sensitivity analyses |  |
| Discussion | | |  |
| Key results | 18 | Summarise key results with reference to study objectives |  |
| Limitations | 19 | Discuss limitations of the study, taking into account sources of potential bias or imprecision. Discuss both direction and magnitude of any potential bias |  |
| Interpretation | 20 | Give a cautious overall interpretation of results considering objectives, limitations, multiplicity of analyses, results from similar studies, and other relevant evidence |  |
| Generalisability | 21 | Discuss the generalisability (external validity) of the study results |  |
| Other information | | |  |
| Funding | 22 | Give the source of funding and the role of the funders for the present study and, if applicable, for the original study on which the present article is based |  |
